# 

**Published:** 1907-05

**Authors:** 


Sixty-second Year.

Vol. LXII. MAY, 1907. No. 10

### BUFFALO MEDICAL JOURNAL

Established 1845 by AUSTIN FLINT, M. D.

INDEXED

### NOTICE OF REMOVAL

The Editorial Offices of this Journal have been removed to Delaware Court, 238 Delaware Avenue, where all communications, correspondence, exchanges and books for review should be addressed

*William Warren Potter.*

